# Sagittal Balance Parameters after Anterior Cervical Discectomy with Spondylodesis and Arthroplasty Using Endocarbon Endoprosthesis: Results of Randomized Study

**DOI:** 10.17691/stm2022.14.4.06

**Published:** 2022-07-29

**Authors:** A.S. Eliseev, A.E. Bokov, S.G. Mlyavykh

**Affiliations:** Assistant, Department of Traumatology, Orthopedics and Neurosurgery named after M.V. Kolokoltsev; Privolzhsky Research Medical University, 10/1 Minin and Pozharsky Square, Nizhny Novgorod, 603005, Russia; Head of the Department of Traumatology, Orthopedics and Neurosurgery named after M.V. Kolokoltsev; Head of the Department of Oncology and Neurosurgery, Traumatology and Orthopedics Institute; Privolzhsky Research Medical University, 10/1 Minin and Pozharsky Square, Nizhny Novgorod, 603005, Russia; Associate Professor, Department of Traumatology, Orthopedics and Neurosurgery named after M.V. Kolokoltsev; Privolzhsky Research Medical University, 10/1 Minin and Pozharsky Square, Nizhny Novgorod, 603005, Russia

**Keywords:** arthroplasty, anterior cervical discectomy, spondylodesis, ACDF, CTDA, sagittal balance, Endocarbon

## Abstract

**The aim of the study** was to examine the effect of cervical segment mobility on spinal sagittal balance parameters after cervical total disc arthroplasty (CTDA) and anterior cervical discectomy and fusion (ACDF) using the first domestic intervertebral disc endoprosthesis.

**Materials and Methods:**

The randomized prospective study included 98 patients (48 with CTDA, 50 with ACDF). Implants used: intervertebral disc endoprosthesis or intervertebral fusion cage (Endocarbon; NPP “MedInzh”, Russia).

Total cervical mobility and range of motion in the target and adjacent vertebral motion segments were studied by functional radiography before surgery, at an early postoperative period (within 3 days), and 3, 6, and 12 months after the intervention.

Values of cervical lordosis (CL, °), cervical sagittal vertical alignment (cSVA, mm), and first thoracic vertebra slope (T1 slope, °) were determined by using spinal radiography. Surgimap V2.2 software (Nemaris, USA) was used for measurements.

**Results:**

When comparing changes of overall cervical mobility at different time intervals, statistically significant differences were obtained in ACDF group (p=0.001). When comparing this parameter between ACDF and CTDA, a statistically significant difference was found only at the early postoperative period (p=0.004).

In CTDA group, the range of motion increased at the operated segment (p=0.001) and decreased at the caudal segment (p=0.002). In ACDF group, no motion was observed at the operated segment (p=0.001) and the range of motion increased at adjacent segments (p=0.001). A statistically significant difference between ACDF and CTDA was obtained only at the operated (p=0.001) and caudal segments (p≤0.002).

Correlation analysis showed no dependence between range of motion influence and regional/global balance values (p>0.5).

The intergroup comparison of cervical lordosis (CL) values revealed a statistically significant difference after 6 (p=0.001) and 12 (p=0.001) months. The best results were obtained at ACDF group towards lordosis increase (p=0.001). The relationship between cervical lordosis and arthroplasty of segments C5–C6, C6–C7 (p=0.003; ρ=0.41) was determined using correlation analysis. The correlation between CL and ACDF (p=0.001; ρ=0.72) was also established.

cSVA comparison between groups showed no difference at preoperative period (p=0.215), 6 (p=0.20) and 12 (p=0.425) months after surgery. cSVAs at both groups were equally close to normal values.

T1 slope changes before and 12 months after surgery were statistically significant at ACDF (p=0.008) and CTDA (p=0.001) groups. T1 slope values comparison between ACDF and CTDA shows statistically significant difference after 12 months (p=0.003). T1 slopes were equally close to normal values 1 year after surgical treatment.

**Conclusion:**

Over a 12-month observation period, the segmental range of motion was found to have no effect on changes of regional and global balance of the cervical spine. No influence was confirmed of range of motion on adjacent level syndrome development — the syndrome was diagnosed in none of the cases.

This study demonstrated the effectiveness of arthroplasty using an Endocarbon endoprosthesis in improving cSVA and T1 slope values, but no significant improvement of CL values after treatment compared to ACDF group.

## Introduction

Currently, surgical treatment of degenerative spine pathology pays much attention to sagittal profile parameters, as their influence on recovery of vital functions and quality of patient’s subsequent life was proven. Neurological impairment occurs when certain spinal parameters significantly deviate from normal values: cervical lordosis (CL, °), first thoracic vertebra slope (T1 slope, °), cervical sagittal vertical alignment (cSVA, mm) [1]. Decreased cervical lordosis correlates with neck pain severity and is considered one of the factors aggravating cervical myelopathy course [2-4]. In this regard, an important neuroorthopedic component of degenerative pathology surgery of cervical spine is the preservation or restoration of its lordosis and the balanced position of the head and neck in relation to spine and pelvis underlying parts [5, 6].

Anterior cervical discectomy and spondylodesis (ACDF) is the most common surgery for spondylogenic radiculopathy and myelopathy. The positive effect of this technique on restoration of sagittal balance parameters is proven. An alternative technique is cervical total discectomy and arthroplasty (CTDA), which has an advantage over ACDF in preserving segmental mobility. CTDA method of implantation and fixation has a number of special features during installation due to endoprostheses’ technical heterogeneity [2, 3]. This study aims to evaluate the effectiveness of ACDF and CTDA techniques to restore sagittal balance parameters of the cervical spine.

**The aim of the study** was to examine the effect of cervical segment mobility on the parameters of spinal sagittal balance after cervical total disc arthroplasty and anterior cervical discectomy and fusion using the first domestic intervertebral disc endoprosthesis.

## Materials and Methods

**Figure 1.**
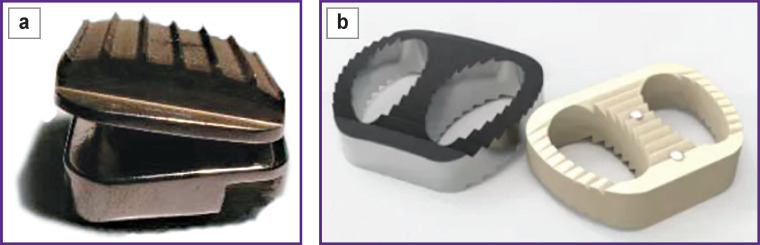
Endocarbon intervertebral disc endoprosthesis (a) and Endocarbon intervertebral fusion cage (b)

A randomized prospective study included 98 patients (48 with CTDA, 50 with ACDF) with symptoms of compressive cervical spondylotic myelopathy and/ or radiculopathy. The method of surgical treatment — ACDF or CTDA — was chosen for each patient using the closed envelope method. Implants used: intervertebral disc endoprosthesis or intervertebral fusion cage (Endocarbon; NPP “MedInzh”, Russia) (Figure 1) which meet necessary safety and effectiveness criteria. The surgical technique was not different from common practice.

Inclusion criteria: 18–80 years of age; pathology ICD-10 diagnosis codes: M42, M48, M50, M53, M54, M99; presence of clinical manifestation of cervical myelopathy and radiculopathy and ineffectiveness of conservative treatment for at least 6 weeks; CT and MRI signs of degenerative-dystrophic changes of one or two vertebral-motor segments (VMS) of the cervical spine with formation of herniated discs and/or their instability and with central and/or unconforaminal stenosis accompanied by clinical compression of the spinal cord or its roots and/or severe pain syndrome in one or two VMSs; written informed consent to participate in the study after receiving all information.

Exclusion criteria: CT and/or MRI signs of degenerative-dystrophic changes of more than two VMSs of the cervical spine; previous surgical interventions on the cervical spine; history of cervical vertebral fracture; concomitant chronic infectious or tumor diseases; pregnancy; marked hypertrophy of facet joints with significant subarticular bone erosions; joint gaps narrowing exceeding 1.7 mm according to CT scan.

Pfirrmann intervertebral disc degeneration grade 5 was not a criterion for exclusion.

The study was approved by the Ethical Committee of Privolzhsky Research Medical University (Nizhny Novgorod, Russia) and was conducted according to the Helsinki Declaration (2013).

All surgeries were performed by surgeons with more than 10-years’ experience. Intraoperative implant position was controlled using Arcadis Varic C-arms (Siemens, Germany) or Vision FD Vario 3D (Ziehm, Germany).

### Surgical technique

The patient’s position on the operating table is supine, the cervical spine in neutral position without lordosis strengthening — for CTDA, extending the cervical spine to strengthen the lordosis — for ACDF. To exclude arbitrary rotation, the head was fixed to the operating table with a self-adhesive hypoallergenic plaster; when the radiographic visualization of the lower cervical segments was limited, the patient’s upper brachial girdle and arms were slightly pulled distally. Classic Cloward approach to left anterior cervical spine was performed. After radiographic identification of the intervention level, a Caspar vertebral body distractor and neck soft tissue retractor were installed. After resection of the anterior parts of the intervertebral disc, total microsurgical discectomy was performed. The segment height was restored using a vertebral body distractor. Then, osteophytes (if any) resection, posterior longitudinal ligament, and unilateral or bilateral uncoforaminotomy were performed. After decompression stage completion, endoprosthesis or cage size was selected successively installing appropriate templates. CTDA template satisfactory size was confirmed by its tight fit to adjacent vertebral bodies, simultaneous central position along interbody space horizontal and vertical axes with interarticular gap size of *articulatio zygapophysialis* not exceeding adjacent joints similar parameter. Criteria for ACDF: central position along interbody space horizontal and vertical axes, template tight fit to vertebral bodies with no signs of implant mobility. The data were assessed by digital radiography in frontal and lateral views after reducing segment distraction. The segment distraction was restored after the endoprosthesis or cage was selected. The endoprosthesis or cage was installed using a special device. The implant adequate position in frontal and sagittal planes was confirmed by radiographs in two projections. Vertebral bodies dorsal boundary and the cervical spine assumed axis of rotation served as landmark to determine installation depth according to X-ray data. The surgical intervention was completed by layered wound closure.

### Radiographic parameters of the cervical spine

Cervical spine general mobility along with range of motion of target and adjacent vertebral motion segments were studied by functional radiography performed before surgery, at the early postoperative period (within 3 days from intervention day) and 3, 6, and 12 months after intervention.

**Figure 2.**
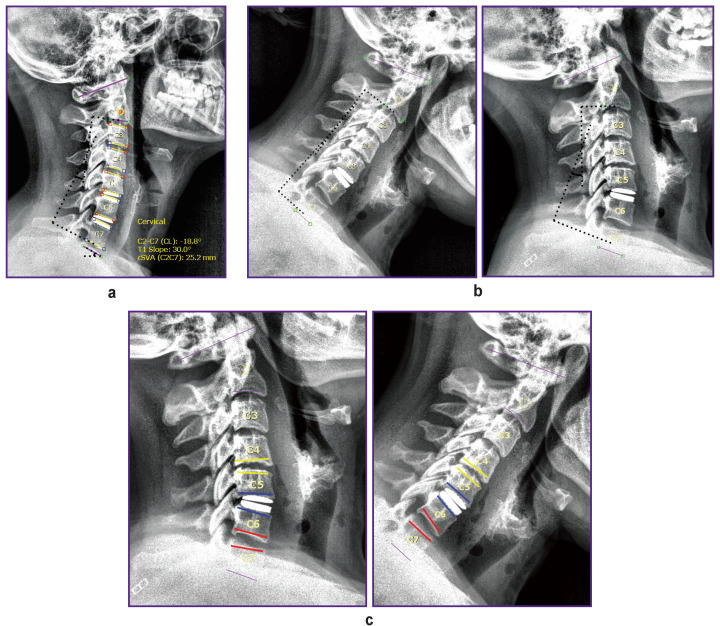
Radiography of the cervical spine and measurement of its parameters using Surgimap V2.2 software: (a) marking of CL, T1 slope, and cSVA parameters in static lateral projection; (b) CL marking in lateral projection to determine cervical spine mobility after arthroplasty at flexion and extension; (c) motion range determination of cervical spine segments after arthroplasty by measuring interbody space angle

Values of cervical lordosis (CL, °), cervical sagittal vertical alignment (cSVA, mm), and first thoracic vertebra slope (T1 slope, °) were determined using spinal radiography (Figure 2). Measurements were performed using Surgimap V2.2 (Nemaris, USA), freely distributed via the Internet.

### Statistical analysis

For statistics processing, IBM SPSS Statistics 23 was used. Data were presented as median and the 25th and 75th percentiles — Me [Q1; Q3]. Shapiro–Wilk test was used to estimate the distribution in excerpts; nonparametric analysis criteria (Mann–Whitney U test, Friedman criterion, Spearman correlation analysis) — in view of distribution different from normal (p≤0.015) [7, 8].

## Results

**Figure 3.**
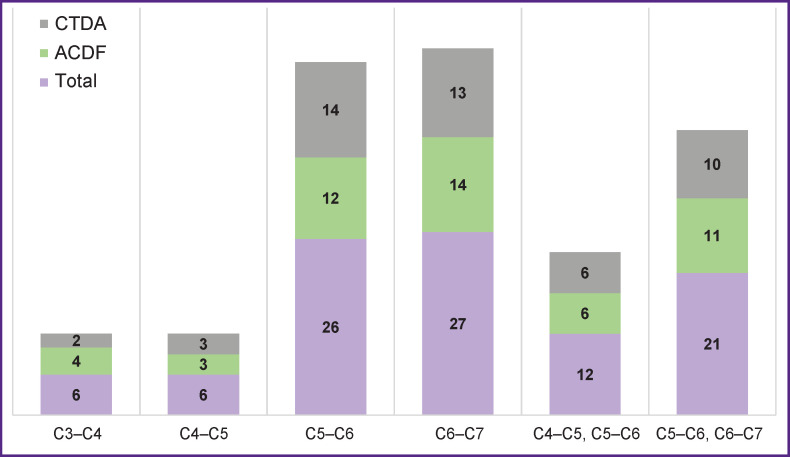
Number of implants installed at different levels

The study included 43 men and 55 women, mean age was 51 [43; 55] years, BMI — 26 [23.96; 29.41]. See Figure 3 for the number of implants installed at different levels.

**Figure 4.**
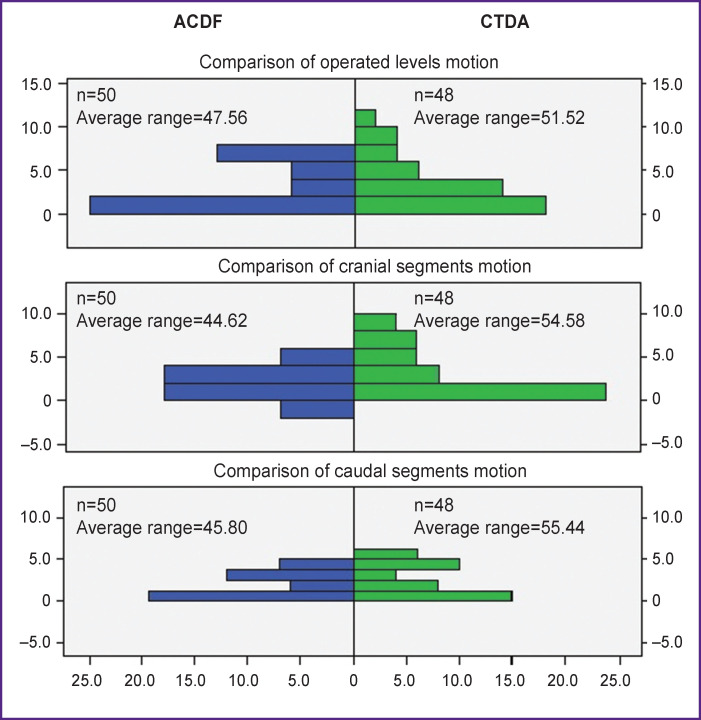
Graphical interpretation of Mann–Whitney analysis for different levels of segmental range of motion

Comparison of range of motions in operated (p=0.64), adjacent cranial (p=0.71), and caudal segments (p=0.74) showed no significant difference between groups at the preoperative stage (Figure 4).

When comparing changes of total cervical mobility at different intervals, no statistically significant differences were found in CTDA group (p=0.067), but statistically significant downward differences were obtained in ACDF group (p=0.001) (Table 1). However, a more detailed overall mobility study between ACDF and CTDA groups revealed a statistically significant difference only at the early postoperative period (p=0.004).

**Table 1:** Values of segment range of motion and cervical spine mobility, Me [Q1; Q3]

Parameters	Time reference point	ACDF (n=50)	CTDA (n=48)	p-independent for values variables
Range of motion of cranial segments (°)	Before surgery	1.70 [1.15; 10.0]	1.91 [1.06; 8.10]	0.71
	Within 3 days after surgery	2.10 [0.60; 3.10]	2.42 [1.22; 4.85]	0.82
	After 3 months	2.20 [1.72; 3.75]	2.60 [1.52; 4.67]	0.76
	After 6 months	2.30 [1.64; 3.78]	2.59 [1.67; 4.62]	0.45
	After 12 months	4.40 [3.77; 5.23]	3.10 [2.82; 6.02]	0.16
p-values for dependent variables		0.001	0.001	—
Range of motion of operated segments (°)	Before surgery	2.70 [0.70; 6.73]	2.85 [0.90; 5.82]	0.64
	Within 3 days after surgery	0.40 [0.22; 0.62]	4.20 [3.02; 6.17]	0.001
	After 3 months	0 [0; 0]	4.95 [2.15; 8.32]	0.001
	After 6 months	0 [0; 0]	5.95 [3.52; 9.07]	0.001
	After 12 months	0 [0; 0]	5.65 [3.12; 8.05]	0.001
p-values for dependent variables		0.001	0.001	—
Range of motion of caudal segments (°)	Before surgery	2.20 [0.77; 3.31]	2.45 [0.85; 4.81]	0.71
	Within 3 days after surgery	3.80 [2.10; 5.15]	1.15 [0.60; 3.87]	0.001
	After 3 months	6.10 [2.71; 8.15]	1.40 [0.52; 3.05]	0.001
	After 6 months	6.30 [2.74; 7.30]	2.01 [1.21; 3.02]	0.001
	After 12 months	7.20 [4.60; 8.02]	2.60 [1.24; 3.30]	0.002
p-values for dependent variables		0.001	0.002	—
Cervical spine mobility (°)	Before surgery	14.20 [11.45; 23.37]	15.10 [9.25; 23.40]	0.37
	Within 3 days after surgery	10.50 [8.07; 14.67]	20.20 [11.90; 26.0]	0.004
	After 3 months	14.30 [10.51; 22.92]	15.20 [14.90; 24.37]	0.94
	After 6 months	14.90 [11.82; 20.22]	15.80 [11.67; 19.65]	0.91
	After 12 months	15.70 [13.40; 20.67]	16.30 [13.45; 21.02]	0.90
p-values for dependent variables		0.001	0.067	—

**Figure 5.**
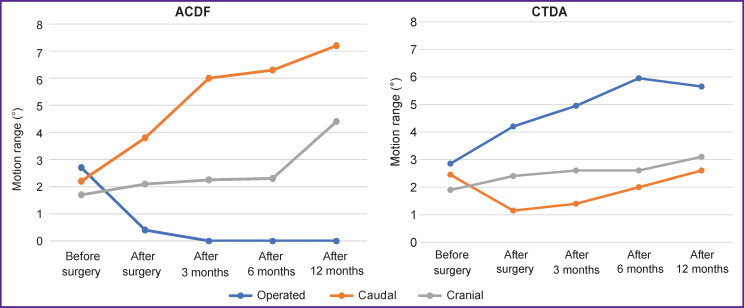
Plots of changes of cervical segment range of motion after ACDF and CTDA at different time intervals

Comparison of range of motion preoperative values with changes at the postoperative stage, after 3, 6, and 12 months revealed statistically significant differences for both ACDF and CTDA groups (Figure 5, see Table 1). In CTDA group, range of motion was noted to increase in the operated segment and to decrease in the caudal segment. In ACDF group, no motion was observed in the operated segment, but the range of motion increased in adjacent segments. A more detailed examination of range of motion differences between ACDF and CTDA yielded a statistically significant difference in operated segments (p=0.001), caudal segments (p≤0.002), but no significant changes in cranial segments (p>0.16).

Correlation analysis showed no dependence between range of motion influence and regional/global balance values (p>0.5).

**Figure 6.**
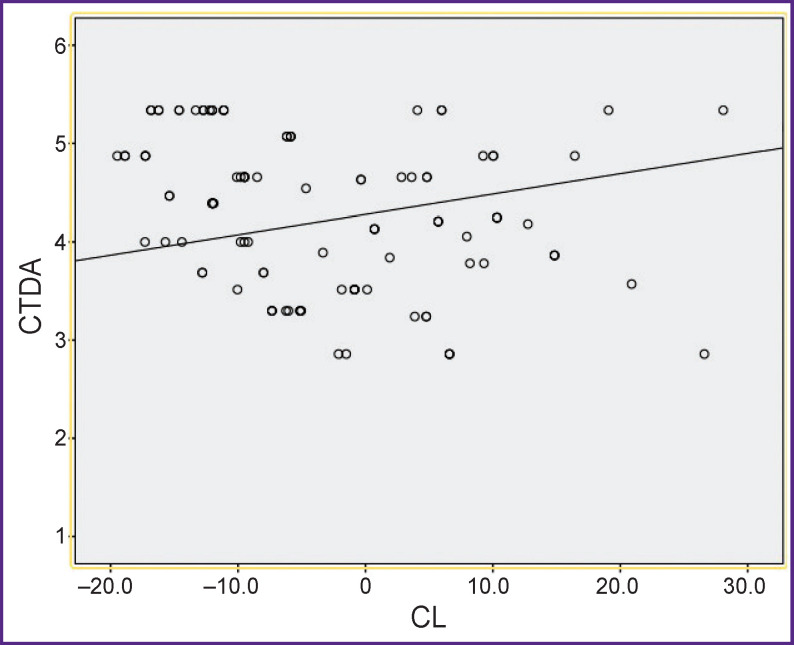
Correlation plot between CL and CTDA at segments C5–C6–C7 level

Table 2 shows the values of sagittal balance parameters for the cervical spine. The intergroup comparison of cervical lordosis (CL) values showed no difference preoperatively (p=0.051) but revealed a difference after 6 (p=0.001) and 12 (p=0.001) months. Assessment of changes dynamics before and after treatment showed a statistically significant difference in ACDF group (p=0.001) toward lordosis increase, but this difference was not noted in CTDA group (p=0.092). However, the relationship between cervical lordosis and arthroplasty of two segments, C5–C6 and C6–C7 (p=0.003; ρ=0.41) (Figure 6) was determined using correlation analysis. The correlation between CL and ACDF (p=0.001; ρ=0.72) was also established.

**Table 2:** Values of sagittal balance parameters for the cervical spine, Me [Q1; Q3]

Parameters	Time reference point	ACDF (n=50)	CTDA (n=48)	for p-independent values variables
CL (°)	Before surgery	6.9 [–12.2; 2.1]	5.4 [–3.78; 10.6]	0.051
	After 6 months	–15.5 [–18.1; –13.8]	2.5 [–10.6; 10.9]	0.001
	After 12 months	–14.4 [–17.3; –12.2]	–8.7 [–9.5; 4.9]	0.001
p-values for dependent variables		0.001	0.092	—
cSVA (mm)	Before surgery	22.3 [16.8; 30.8]	21.2 [13.6; 25.8]	0.215
	After 6 months	20.7 [15.9; 24.8]	20.3 [15.9; 23.6]	0.20
	After 12 months	20.1 [17.6; 21.9]	20.4 [17.7; 22.9]	0.425
p-values for dependent variables		0.006	0.006	—
T1 slope (°)	Before surgery	17.9 [17.1; 19.1]	27.3 [25.7; 31.3]	0.540
	After 12 months	24.1 [23.0; 27.2]	24.1 [23.0; 31.3]	0.003
p-values for dependent variables		0.008	0.001	—

cSVA changes dynamics before and after treatment was statistically significant in both ACDF (p=0.006) and CTDA groups (p=0.006). Comparison of this parameter between the groups showed no statistically significant difference at the preoperative stage (p=0.215), 6 (p=0.20) and 12 (p=0.425) months after surgery (see Table 2). A correlation was found between cSVA and T1 slope (p=0.011; ρ=0.56) and between cSVA and CL (p=0.012; ρ=–0.31).

The comparative evaluation of T1 slope changes before and 12 months after surgery shows a statistically significant difference in both ACDF (p=0.008) and CTDA groups (p=0.001). When comparing T1 slope values between ACDF and CTDA, a statistically significant difference was not obtained at the preoperative stage (p=0.54), but was detected during examination after 12 months (p=0.003) with T1 slope increase in ACDF group. Correlation between T1 slope and CL (р=0.016; ρ=0.44) was found.

## Discussion

In order to recreate normal biomechanics and preserve the mobility of all cervical spine segments, arthroplasty technique was developed. The importance of studying cervical segments mobility lies in its possible influence on development of adjacent level syndrome. Degenerative changes progression is directly related both to segmental motion and cervical spine motion overall volume [9] and to the effect of these movements on intradiscal pressure [10] which causes morphological changes of the intervertebral disc [11, 12].

This study showed increased range of motion at the operated segment in CTDA group, which is not surprising since mobility preservation after ACDF is caused either by incorrectly performed surgery or by pseudoarthrosis development [13]. More attention should be paid to range of motion changes in adjacent segments due to possible development of adjacent level syndrome and, as a consequence, the need for repeated surgical intervention [14, 15]. Our study demonstrated increased amplitude in adjacent segments after ACDF and decreased amplitude in the caudal segment and no change in the cranial segment in CTDA group. Similar results were obtained during experimental studies [9, 10]. The increased amplitude of adjacent segments may later serve as a factor of adjacent level syndrome development. Assessment of total cervical mobility revealed a difference between the groups only at the early postoperative period; no changes were found at other time points, which suggests compensation of movements due to load redistribution in adjacent segments since total cervical mobility after spondylodesis is not different from the arthroplasty group.

The relationship between some parameters of cervical spine sagittal balance is known. Low lordosis (CL) affects intensity increase of neck pain, and Th1 vertebra slope is directly related to lordosis magnitude. T1 slope importance, in addition to its influence on cervical lordosis, lies in its influence on the global sagittal line (SVA), thoracic kyphosis, and a Th1 vertebra slope value larger than 32° is a predictor of sagittal spine and pelvis disorders [1, 16, 17]. T1 slope is variable, so it is possible to change it altering cervical lordosis and other parameters.

This study also shows the relationship between Th1 slope and cervical lordosis. Lordosis improvement was detected in ACDF group; no significant improvement was observed in CTDA group. This can be explained by different approaches at the stage of laying the patient’s neck: extension of the cervical region when performing anterior fusion and maintaining a neutral position when performing arthroplasty, also by implant configuration which determines the objectives of these surgical treatment methods as the cage has beveled edges at contact areas with closing plates of adjacent vertebral bodies in anteverted direction to get better fixed in the segment and to create segmental lordosis, while the endoprosthesis lacks these design features due to its mobility. However, publications are available describing lordosis improvement after arthroplasty, but only at endoprosthetic replacement of two or more levels [18]. Our study showed that arthroplasty of lower cervical segments at two levels (C5–C6, C6–C7) improves lordosis compared to single-level arthroplasty.

Studying cSVA importance, various authors reported evidence of life quality worsening of adult patients with a more than 40 mm increase of anterior sagittal displacement [17, 19]. The results of our study include data on cSVA improvement in both groups with no significant difference between them.

## Conclusion

Data were obtained on no influence of segmental range of motion over changes of cervical spine regional/ global balance during the 12-month observation period. No confirmation was obtained in our study of range of motion influencing the development of adjacent level syndrome — this was not diagnosed in any of the cases, which may be due to insufficient observation time for its manifestation.

Effectiveness of arthroplasty was demonstrated using an Endocarbon endoprosthesis to improve cSVA and T1 slope values, but without obtaining significant improvement of CL values after treatment compared to ACDF group.
